# Case of Biliary Calculi

**Published:** 1868-05

**Authors:** R. Dunlap

**Affiliations:** Waukesha, Wis.


ARTICLE XVII.

### CASE OF BILIARY CALCULI.

By R. DUNLAP, M.D., Waukesha, Wis.

Was called, on Saturday, March 14, to see Mr. John H. Foster, in consultation with Dr. Steele. Found he had been taken sick on Wednesday, the 11th, with bilious colic; had been in great pain through the bowels, attended with vomiting large quantities of bile for three days, without any passage from the bowels. The pain had left, but there was vomiting of a dark brown color every time any fluid was taken, but in small quantities and without any great effort. There had not been any cramping or straining. Pulse 97, and no fever or inflammation; tongue of a thin brown color; skin cool and a little moist; some soreness and tenderness over the region of the stomach.

Dr. Steele first gave an emetic, followed by submur. hyd. and opii in small doses, to the amount of about 20 grs., and applied counter-irritants to the abdomen; had used injections freely, with operations from the bowels but twice. The first injection came away as it was given, and in about an hour he had a large passage (on Wednesday) of dark, hard fæces, looking like small worms. The other injection came away as given. I recommended to follow up the submur. hyd., in about 20 gr. doses every 4 hours, and applied croton oil to the stomach.

Sunday, added large doses of opium to the submur. hyd. Monday, Dr. Garner, of Milwaukee, was called, and advised submur. hyd. 20 grs., strychnine $\frac{1}{16}$ gr., prussic acid, and cyanide of potassa. Tuesday, used a warm bath about the middle of the day, and immediately after gave 25 grs. submur. hyd. and 2 drops croton oil. About 7 o'clock he had a passage from the bowels, which continued moderately during the night and fore part of the next day, accompanied with some bile. Vomiting had continued the greater part of the time, but not so severe, in smaller quantities, and not so frequently. I forgot to say that on Saturday, the first day I was called, I advised small pieces of ice to be held in the mouth, which was continued the whole time, with a very pleasant feeling, if not with any success. The pulse had remained, all the time, from 80 to 90.

Wednesday, he felt exceedingly well; but very little vomiting; skin natural; pulse 110 and soft. About 7 o'clock in the evening, a sudden change took place. He began to sink; a cold, clammy sweat came on; his pulse was almost imperceptible; great distress for breath; and restlessness. Brandy was freely given, but did no good. Thursday morning, I was sent for. Finding him in the above state, I gave brandy and quinine, which was used all day, accompanied with rubbing with stimulating washes and using all the external applications we could think of, but all to no effect. He breathed his last at 9½ o'clock, as if he was going asleep.

*Post Mortem.*—Found the bowels empty, excepting a little fluid; saw where the stricture had been; some little mortification; the liver somewhat enlarged; and in the gall-bladder were 142 stones, from the size of a hickory nut to that of a small pea, all weighing one ounce.

Mr. Foster came to see me last winter, concerning his back, complaining of a fluttering just above the small of the back. He had no pain, but that disagreeable fluttering. He had a rupture for many years, but it had healed up, and he had not worn a truss for three years. This fluttering troubled him during his sickness.

